# Extensive myeloid sarcoma presenting with esophageal compression and dysphagia as the initial manifestation

**DOI:** 10.1097/MD.0000000000046337

**Published:** 2026-05-12

**Authors:** Haoyu Li, Liting Dai, Xiao Wang, Yanbo Yu

**Affiliations:** a Department of Gastroenterology, Qilu Hospital of Shandong University, Jinan, Shandong, China; b Department of Pathology, Qilu Hospital of Shandong University, Jinan, Shandong, China.

**Keywords:** diagnostic challenge, dysphagia, esophageal mass, immunohistochemistry, myeloid sarcoma

## Abstract

**Rationale:**

Myeloid sarcoma (MS) is an extramedullary tumor of immature myeloid cells that can precede or accompany acute myeloid leukemia (AML). Mediastinal/retroperitoneal involvement causing esophageal compression is rare and prone to misdiagnosis.

**Patient concerns:**

A 67-year-old woman presented with 20 days of progressive dysphagia.

**Diagnoses:**

Contrast-enhanced Computed tomography showed infiltrative soft-tissue masses in the posterior mediastinum, para-aortic region, and bilateral chest/abdominal walls, encasing the esophagus and major vessels; endoscopy confirmed extrinsic esophageal stenosis. EUS-FNA was nondiagnostic for small-cell carcinoma. Core biopsy with high-power review revealed predominantly medium-to-large blasts with focal granulocytic maturation. Immunophenotype supported myeloid lineage: MPO diffuse positive; CD117 positive; focal CD34 and CD99; weak-to-focal CD45/LCA; negative B-cell (CD19, CD20, CD79a), T-cell (CD2, CD3, CD5, CD7), and epithelial markers (CK AE1/AE3, CK19). Ki-67 proliferation rate was 40%–50%. Peripheral blood counts were unremarkable for blasts or cytopenias; bone marrow aspirate/biopsy/flow showed no diagnostic evidence of AML. Final diagnosis: MS with extrinsic esophageal compression.

**Interventions:**

AML-type systemic chemotherapy with the HAA regimen (homoharringtonine 3 mg d1–7, aclarubicin 20 mg d1–7, cytarabine 0.2 g d1–7; q3 weeks) plus standard supportive care.

**Outcomes:**

Dysphagia improved during the first cycle. Interval imaging demonstrated reduction of mediastinal/retroperitoneal soft-tissue bulk and improved esophageal lumen consistent with disease control. No radiotherapy or esophageal stenting was required. On follow-up, disease remained controlled without transformation to AML.

**Lessons:**

Infiltrative mediastinal/retroperitoneal MS may present with esophageal obstruction and mimic lymphoma or carcinoma. High-index suspicion, targeted biopsy with high-power morphology, and a focused immunohistochemical panel are critical for timely diagnosis and treatment initiation.

## 1. Introduction

Myeloid sarcoma (MS), an extramedullary tumor of immature myeloid cells, typically involves lymph nodes, skin, or soft tissues. Involvement of the mediastinum and retroperitoneum is exceedingly rare, with few reports of esophageal compression. MS may precede, coincide with, or relapse as acute myeloid leukemia, and its diagnosis is often delayed due to mimicry of lymphoma or carcinoma. We describe a case of primary MS causing dysphagia through extensive thoracic and abdominal infiltration, emphasizing its diagnostic complexity and role in expanding differentials for esophageal masses.

## 2. Case presentation

**Figure 1.**
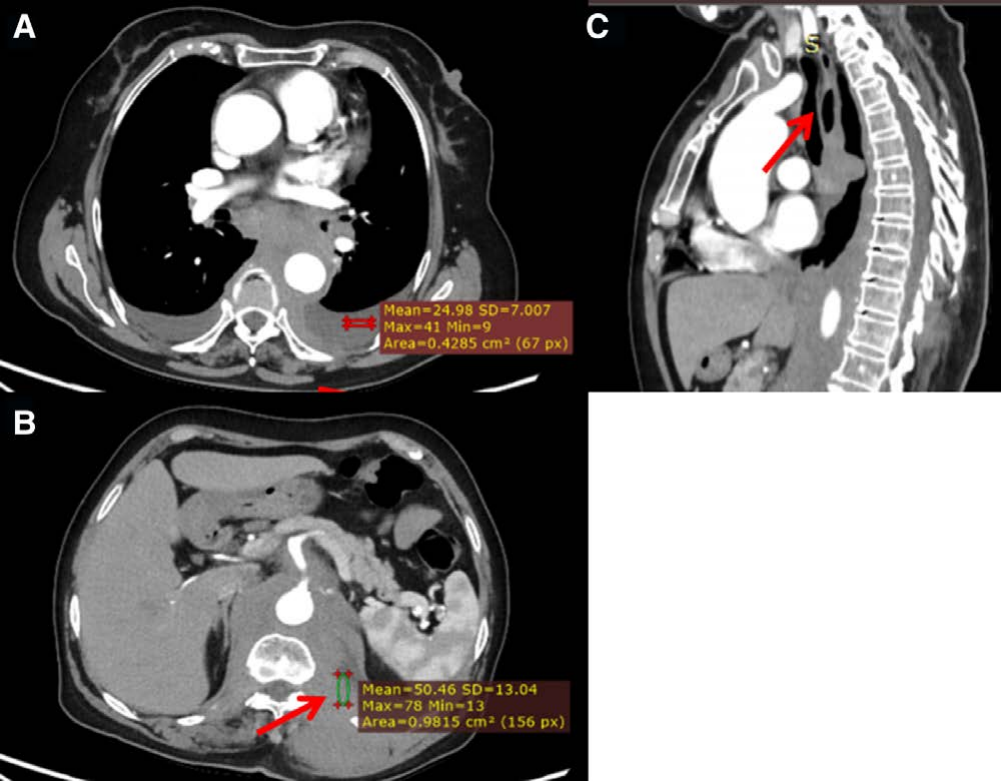
Contrast-enhanced CT images de1monstrating extensive infiltrative lesions (A) middle and posterior mediastinum; (B) celiac trunk and retroperitoneum space; (C) esophagus. CT = computed tomography.

**Figure 2.**
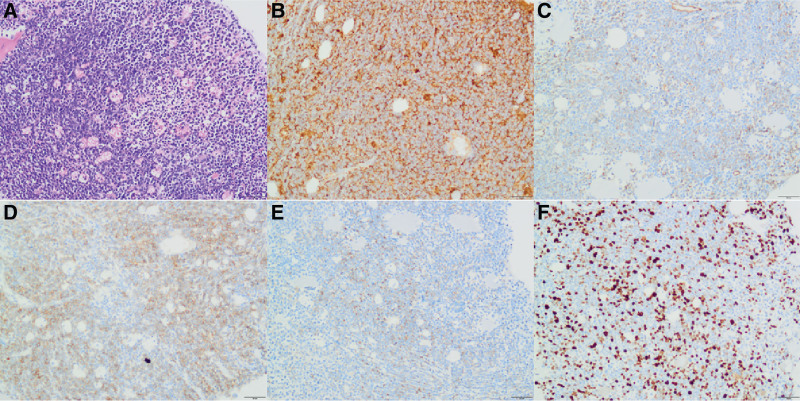
Histopathological and immunohistochemical findings. (A) Initial H&E stain suggested lymphoid hyperplasia. Subsequent IHC revealed; (B) marked diffuse immunoreactivity for MPO; (C) Focal CD34 positivity; (D) CD117 positivity; (E) Focal CD99 positivity; (F) Ki-67 positivity in approximately 40%–50% of tumor cells. IHC = immunohistochemistry.

A 67-year-old woman presented with a 20-day history of progressive dysphagia. Initial contrast-enhanced CT revealed infiltrative soft-tissue masses involving the posterior mediastinum, para-aortic region, and bilateral chest/abdominal walls, encasing critical structures including the esophagus, left renal artery, and superior mesenteric artery. Endoscopy confirmed esophageal stenosis with extrinsic compression. Endoscopic ultrasound (EUS) identified an adjacent heterogeneous hypoechoic lesion. EUS-guided fine-needle aspiration (EUS-FNA) cytology demonstrated atypical cells negative for small-cell carcinoma markers. Repeat CT scan continued to show persistent infiltrative lesions, radiologically suggestive of lymphoproliferative disease. Subsequent CT-guided biopsy of a chest wall lesion initially suggested lymphoid hyperplasia (Fig. 1). However, definitive immunohistochemical analysis revealed a distinct profile: MPO diffuse positive; CD117 positive; focal CD34 and CD99; weak-to-focal CD45/LCA; negative B-cell (CD19, CD20, CD79a), T-cell (CD2, CD3, CD5, CD7), and epithelial markers (CK AE1/AE3, CK19). Ki-67 proliferation rate was 40%–50% (Fig. 2). This constellation of morphologic and immunohistochemical features established the diagnosis of MS.

Given the symptomatic esophageal obstruction, the patient promptly received AML-type systemic therapy with the HAA regimen (homoharringtonine 3 mg days 1–7, aclarubicin 20 mg days 1–7, cytarabine 0.2 g days 1–7; every 3 weeks). Standard supportive care was provided according to institutional practice. Dysphagia improved within the first treatment cycle, permitting advancement of oral intake. Interval imaging demonstrated reduction in the bulk of the mediastinal/retroperitoneal masses with improvement in the esophageal lumen, consistent with disease control; no radiotherapy or esophageal stenting was required. The regimen was overall tolerated, and on subsequent follow-up the disease remained controlled without transformation to acute myeloid leukemia (Table 1).

**Table 1:** Timeline of clinical events.

Relative to presentation	Event
−20 d	Symptom onset: progressive dysphagia.
Day 0 (initial evaluation)	Contrast-enhanced CT: infiltrative soft-tissue masses in posterior mediastinum, para-aortic region, and bilateral chest/abdominal walls, encasing the esophagus and major vessels. Endoscopy: extrinsic esophageal stenosis. EUS: adjacent heterogeneous hypoechoic lesion.
+3–7 d	EUS-FNA cytology: nondiagnostic for small-cell carcinoma.
+1–2 wk	Repeat CT: persistent infiltrative lesions, radiologically suggesting lymphoproliferative disease.
+2–3 wk	CT-guided core biopsy of chest wall lesion: initially read as lymphoid hyperplasia; definitive IHC shows MPO diffuse positive, CD117 positive, focal CD34 and CD99, weak-to-focal CD45/LCA; negative B-cell (CD19, CD20, CD79a), T-cell (CD2, CD3, CD5, CD7), and epithelial markers (AE1/AE3, CK19). Morphology: predominantly medium-to-large blasts with high N:C ratios, fine chromatin, inconspicuous nucleoli, scant–moderate cytoplasm; focal granulocytic maturation without convincing promyelocytic morphology. Ki-67 manually re-verified ≈ 40–50%. → Final diagnosis: myeloid sarcoma with extrinsic esophageal compression.
Same period	Baseline systemic work-up: peripheral blood counts without blasts/cytopenias; bone marrow aspirate/biopsy/flow with no diagnostic AML; pelvic/gu imaging without suspicious lesions; symptom-driven neuro review negative for CNS involvement.
+3–4 wk	Initiated AML-type HAA regimen: homoharringtonine 3 mg d1–7, aclarubicin 20 mg d1–7, cytarabine 0.2 g d1–7; q3 weeks, with standard supportive care.
During cycle 1	Dysphagia improved; oral intake advanced.
Post-cycle 1 (interval assessment)	Imaging: decreased mediastinal/retroperitoneal soft-tissue bulk and improved esophageal lumen – consistent with disease control. No radiotherapy or esophageal stenting required.
Follow-up (ongoing)	Disease remains controlled; no transformation to AML.

AML = acute myeloid leukemia, CNS = central nervous system, EUS-FNA = endoscopic ultrasound–guided fine-needle aspiration, HAA = homoharringtonine (d1–7) + aclarubicin (d1–7) + cytarabine (d1–7), q3w, IHC = immunohistochemistry, MPO = myeloperoxidase.

Given that MS can precede or accompany acute myeloid leukemia or other myeloid neoplasms, we documented a baseline hematologic and systemic evaluation to exclude concomitant disease. Peripheral blood counts with differential were obtained (WBC 4.56 × 10^9^/L, neutrophils 52.10%, lymphocytes 36.70%, monocytes 7.10%, hemoglobin 12.8 g/dL, platelets 237 × 10^9^/L), showing no circulating blasts and no overt cytopenias or monocytosis; LDH was not obtained at presentation. Bone marrow assessment – including aspirate cytology, trephine biopsy, and flow cytometry – showed no diagnostic evidence of AML and no significant dysplasia; the blast percentage was not quantified/recorded. To evaluate potential extramedullary “sanctuary” sites, pelvic/genitourinary imaging revealed no suspicious lesions, and a symptom-driven neurologic review found no indications for CNS involvement. Collectively, these findings argue against simultaneous or preceding systemic myeloid disease at presentation.

## 3. Discussion

MS^[1]^ represents an uncommon extramedullary solid tumor composed of immature myeloid cells. Characterized by its low incidence, MS can manifest as an isolated mass lesion in patients without overt hematologic manifestations of leukemia. Critically, these lesions frequently herald the development of acute myeloid leukemia (AML), with progression typically occurring within 11 months, establishing MS as a recognized AML precursor.^[2]^ The most frequent anatomical sites include lymph nodes, skin, and soft tissues.

The diagnosis of isolated MS presents significant challenges in the absence of typical hematologic findings. This difficulty is reflected in the high reported initial misdiagnosis rates, ranging from 47% to 57%.^[3,4]^ Histopathological evaluation by conventional light microscopy often lacks distinctive features and can be further complicated by biopsy site variations. Consequently, MS is frequently misinterpreted as lymphoproliferative disorders, lymphoma, various blastomas, or other sarcomas.^[5,6]^ Definitive diagnosis is therefore heavily reliant on immunohistochemical profiling. Key markers consistently reported as valuable for confirming the myeloid lineage and securing the diagnosis include MPO, CD33, CD34, CD43, and CD117.^[5,7]^

At presentation, cytogenetic and molecular studies were not performed due to limited lesional tissue and prioritization of symptom-relieving therapy; this is acknowledged as a limitation. If additional tissue or bone marrow becomes available or if the disease evolves, we plan to perform conventional karyotype ± targeted FISH and a myeloid-focused next-generation sequencing panel (including NPM1, FLT3, RUNX1, TP53, KMT2A, CEBPA, among others) to refine diagnosis and prognosis and to inform treatment decisions. Additionally, we acknowledge that further immunostains to confirm monocytic differentiation were not performed because no residual lesional tissue remained after the diagnostic work-up. In particular, CD15 and monocytic-lineage markers were planned but were not feasible. This constraint explains the relatively limited immunophenotypic panel reported in the case presentation; nevertheless, the available profile was sufficient to establish MS in the clinical context.

Efforts to reduce diagnostic error and improve patient survival remain critical. In this case, a comprehensive diagnostic evaluation facilitated timely and accurate identification of MS. This prompt diagnosis enabled the initiation of appropriate therapy, correlating with the observed favorable clinical outcome.

## 4. Conclusion

To our knowledge, this case represents the first reported instance of esophageal compromise caused by mediastinal/retroperitoneal MS without bone marrow involvement at presentation, which makes the diagnosis exceptionally challenging. This case illustrates the profound diagnostic challenge it presents, particularly when manifesting as esophageal obstruction. Initial misdiagnosis as lymphoma is common. Definitive diagnosis requires a high-index of suspicion and comprehensive immunohistochemical analysis. Prompt recognition is essential for initiating appropriate therapy, which may prevent progression to AML and improve outcomes. Our findings underscore the importance of including MS in the differential diagnosis of unexplained mediastinal/retroperitoneal masses causing compressive symptoms.

## Acknowledgments

We thank the efforts and contributions of the reported patients and all the clinical staff in this study.

## Author contributions

**Conceptualization**: Haoyu Li, Yanbo Yu.

**Data curation**: Liting Dai, Xiao Wang.

**Funding acquisition**: Yanbo Yu.

**Writing – original draft**: Haoyu Li.

**Writing – review & editing**: Haoyu Li.
